# What Women With Dementia Who Receive Home Care Services Consider Important in Daily Life: A Qualitative Study

**DOI:** 10.1177/23779608261479697

**Published:** 2026-08-17

**Authors:** Simen A. Steindal, Ingebjørg Haugen, Orla Brady, Knut Engedal, Benedicte Sørensen Strøm

**Affiliations:** 1 Faculty of Health Sciences, 87368 VID Specialized University, Oslo, Norway; 2 155319 Lovisenberg Diaconal University College, Oslo, Norway; 3 School of Health Sciences, Aras Moyola, 8799 University of Galway, Galway, Ireland; 4 Norwegian Advisory Unit for Aging and Health, 60512 Vestfold Hospital HF, Tønsberg, Norway; 5 Department of Geriatrics, Oslo University Hospital, Oslo, Norway; 6 Diakonhjemmet Care, Oslo, Norway

**Keywords:** dementia, home-dwelling, home care, nursing, qualitative research, women

## Abstract

**Introduction:**

Most people with dementia in Norway live at home, and maintaining physical, social and spiritual activity remains a basic need. Yet research has focused on women as caregivers rather than women living with dementia. Consequently, more knowledge is needed into what women receiving home care service consider important in daily life to tailor health care services and to understand how these women experience participating in activities.

**Objective:**

The aim of this study was to describe what women with dementia who receive home care service consider important in their daily lives.

**Methods:**

This study employed an exploratory–descriptive design. Data were collected using individual semi-structured interviews with eight older women with dementia with mild to moderate cognitive impairment who received home care service. Data were analysed using manifest qualitative content analysis.

**Results:**

The data analysis identified three categories: The need to be physically active and spend time outdoors, The need to staying socially connected and The need for meaningful activities.

**Conclusion:**

This study provides insight into what women with dementia who receive home care service consider important in their daily lives. The need for meaning in daily life can be met in different ways, and it is important to tailor activities for women with dementia.

## Introduction

The estimated percentage of people with dementia (PwD) living at home ranges from 50–70% in high-income countries to 94% in low- and middle-income countries (Prince et al., 2015). In 2022, 328,245 people in Norway were receiving health care in the home (Helsedirektoratet, 2023), of which two-thirds were diagnosed with dementia (Gjøra et al., 2021).

Living at home can help maintain quality of life (Olsen et al., 2016), and Norwegian citizens residents have a legal right to social and health care, regardless of age, gender, socioeconomic status or other difference (Ministry of Health and Care Services, 2011). The current Norwegian dementia strategy (Norwegian Ministry of Health and Care Services, 2022) aims to assist PwD to live at home for as long as possible to allow older people to live active lives longer and feel safe in their own homes (Meld. St. 24, 2023). However, in general, older people live less active lives due to barriers such as fear, lack of company, unsuitable environment, disabilities, poor balance, fear of injury and depressive symptoms (Hartman et al., 2018). Furthermore, living with dementia can make everyday activities more demanding and may cause people to lose a sense of belonging to their community and disengage from their usual activities (Norwegian Ministry of Health and Care Services, 2022).

Physical activity is particularly important for older PwD (Forbes et al., 2015; McDuff & Phinney, 2015) as physical activity can possibly slow down the progression of cognitive dysfunction (Jia et al., 2019; Livingston et al., 2020; Olsen et al., 2015). In addition, the pleasure of engaging in physical activity is important for staying motivated and engaged throughout life (van der Wardt et al., 2020). However, several barriers, including cognitive impairment, dependence on others, lack of access to dementia-specific exercise programmes, apathy and lack of initiative (Baber et al., 2021), can make it difficult for PwD to participate in physical activities (Hobson et al., 2020; Telenius et al., 2020). Gait and balance impairments may also increase during the course of dementia, which increases the risk of falling (Hoogendijk et al., 2019). In addition to the importance of physical activity, Kratzer et al. (2022) emphasise the need to provide cognitive and social activities for PwD. Cognitive and social activities may even be beneficial in reducing the overall risk of developing dementia (Duffner et al., 2022). Studies also support the importance of including spirituality for PwD to help them find meaning, hope and connection to the past, present and future and in coping with their condition (Beuscher & Beck, 2008; Beuscher & Grando, 2009).

### Review of the Literature

Previous studies have explored the perspectives of PwD regarding how they experienced lived spaces in different contexts, such as living alone and receiving home care services (Førsund et al., 2018). Studies have also investigated PwD’s experience of meaningful activity, their experience of the outdoor environment and the important aspects of their lives (Førsund et al., 2018). A previous study explored the experiences and attitudes of PwD related to living at home. The findings suggested that PwD experienced a vital interconnectedness between their home and their life, identifying their home as a core foundation for life. Progressive dementia disturbed the rhythms of life at home, forcing these people to adapt and change their routines (Fæø et al., 2019). Another study investigated the factors that influenced everyday coping strategies in people with early to intermediate dementia. The findings suggested that coping with everyday life was affected, for example, by challenges in everyday life. Most of the participants wanted to participate in day care services several days a week, make new friends and participate in meaningful activities, which could enhance their coping strategies (Moe et al., 2021).

Ensuring that activities become a central part of the services offered to PwD is very important since this contributes to meaning, effective coping and positive experiences for individuals (Norwegian Ministry of Health and Care Services, 2022). Lack of meaningful activities can increase the risk of being isolated and lonely (Bjørkløf et al., 2019). Previous research reported that people living with dementia expressed the need to stay connected, to be active and participate and to live for the moment (Telenius et al., 2020). Research is available on how people experience and cope with dementia (Sharp, 2019) and on the effects of activities for PwD. There has been a growing recognition that gender influences dementia, and evidence indicates that dementia should be prioritized as a women’s health concern. However, the perspectives of women living with dementia are largely absent, as research has often focused on women as family caregivers of PwD rather than living with dementia (Erol et al., 2016). Therefore, more knowledge is required about what women living with dementia who receive home care service consider important in their daily lives to be able to tailor healthcare services and understand how they experience participating in activities. Consequently, the aim of this study was to describe what women with dementia who receive home care service consider important in their daily lives.

## Methods

### Design

This study employed an exploratory–descriptive design using individual semi-structured interviews. This design is suitable for exploring, describing and understanding the experiences of older women with dementia since little is known about this topic (Hunter et al., 2018). Individual interviews may give older women time to reflect and share experiences that are important to them with the interviewer. The study was reported according to consolidated criteria for reporting qualitative research (Tong et al., 2007) (Supplementary File 1 ).

In 2021, a grant from the Norwegian Health Directorate was awarded to a project aimed at counteracting loneliness and passivity in PwD who receive home care service by offering organised activities. The project was delivered by a home care service in cooperation with volunteers. The participants were older PwD who were recruited from home care services in two city districts in Oslo, Norway. The participants were invited to participate in different social activities every fortnight over a period of six months. Outings such as visiting museums, attending a concert and having a meal together were organised by a group of eight volunteers, always assisted by at least one care staff from the home care facility.

### Inclusion Criteria and Sample

A registered nurse with the home care service acted as a contact for this project and purposefully recruited participants who met the following criteria: (a) had been diagnosed with dementia and had mild to moderate cognitive impairment based on an assessed by the contact person using the Norwegian third revised version of the Mini-Mental State Examination (MMSE-NR3; MMSE-NR3; scores > 10) (Strobel & Engedal, 2021), (b) had the capacity to express their experiences in an interview and provide written informed consent, (c) living at home and receiving home care service, (c) were aged 65 years or older, and (d) had participated in at least one activity organised by the home care service. We chose to include people with mild to moderate cognitive impairment to include participants with diverse experiences to facilitate nuanced and rich data.

We excluded PwD with severe cognitive decline assessed using the MMSE-NR3 (score 1-10) as they often lack the ability to communicate and express their experiences and it would also have been challenging to gain informed consent due to the level of cognitive impairment. After the participants agreed to participate, the first and second authors then provided further information about the study and scheduled time for an interview.

Twelve men and women with dementia who participated in the social activities provided by the home care service were invited to participate in this study by the contact person. Eight women agreed to participate in the study. The mean age was 83 (range: 69–9) years, seven lived alone, one with her husband and the mean MMSE-NR3 score for the seven participants was 16.3 (range: 16–22); one participant refused to complete the MMSE-NR3 (Table 1).

**Table 1.** The Participants

Participants	Gender	Age	Cohabitant	MMSE-NR3
P1	F	86	No	20
P2	F	80	No	17
P3	F	69	No	22
P4	F	82	No	16
P5	F	77	Yes	22
P6	F	89	No	16
P7	F	90	No	18
P8	F	91	No	Rejected

### Data Collection

The semi-structured interviews, lasting between 20 and 50 minutes, were conducted between June and August 2022 in the participants’ homes. The interviews were conducted by the second and last authors, who are female researchers with experience working with PwD and conducting qualitative interviews. Consequently, these authors have extensive experience communicating with PwD. Neither of the interviewers had a personal relationship with any of the participants. A semi-structured interview guide was used to facilitate reflection and dialogue with the participants. The interview guide included the following topics: previous interests and participation in social activities, experiences of participation in activities provided by the home care service and wishes for the future (Supplementary File 2). The interview guide was not piloted because of the limited study population. However, the research team, including researchers with expertise in dementia care, discussed the interview guide several times to ensure clear and relevant questions.

The original aims of the study were to explore the experiences of home-dwelling people living with dementia in terms of their participation in activities before and after the COVID pandemic and their experience of participating in activities provided by the home care services after COVID. However, only women agreed to participate in the study and during the interview, the participants could not retrospectively talk about and describe their experiences before and during COVID. The interviewer carefully and respectfully asked probing questions addressing the intended study aim. However, all the participants kept coming back to sharing their experience of living at home and participating in activities provided by the home care services. All interviews were audio-recorded.

### Ethical Considerations

The study was approved by the Norwegian Agency for Shared Services in Education and Research (ref. No. 907888). As no data were collected with regard to the participants’ health, according to Norwegian law, ethical approval from a regional committee for research ethics was not required. However, the home care service that cooperated in carrying out the study provided ethical approval. The participants received written and verbal information tailored to people with mild to moderate dementia; this information emphasised that participation was voluntary and that they could withdraw from the study without any consequence for their participation in the organised activities or services provided by the home care service. In addition, the written information stated that the interview would be audio recorded. We also emphasised that anonymity and confidentiality would be safeguarded. To protect participant anonymity, we presented the findings carefully to prevent home-care services from identifying individual participants. To promote voluntary participation and avoid undue influence, the contact person who assessed whether potential participants could understand the study information and provide written informed consent was not a member of the research team. Another ethical consideration was revising the study’s original aim: we changed the aim to ensure the study reflected the women’s voices and what mattered most to them.

### Data Analysis

The interviews were transcribed, and the transcripts were checked for accuracy against the audio recordings and analysed using qualitative content analysis on a manifest level (Graneheim & Lundman, 2004; Lindgren et al., 2020), as this study wanted to describe the experiences of older women with dementia.

First, all the authors independently read the interview transcripts several times to gain an overall understanding of the data. Guided by the aim of the study, the first, second and last authors systematically identified meaning units and then condensed and coded the meaning units independently. Then, the codes were grouped into subcategories based on the similarities and differences between the codes. Finally, the subcategories were abstracted and organised into categories on a manifest level. The authors held continuous discussions throughout the analytical process and did not seek consensus. Those with expertise in dementia drew on their knowledge in interpreting the data, while the other authors asked whether there could be alternative interpretations. These discussions encouraged reflection and competing interpretations and helped the authors move beyond their pre-understandings, resulting in several revisions of codes and categories.

## Results

The data analysis identified three categories of things that women with dementia who received homecare services consider important in their daily lives. The data analysis identified three categories: The need to be physically active and spend time outdoors, The need to staying socially connected and The need for meaningful activities. These categories and subcategories are presented in Table 2**.**

**Table 2.** Categories and Subcategories Identified From the Analysis

Categories	The need of being physically active and spend time outdoors	The need to stay socially connected	The need for meaningful activities
Subcategories	The need of maintaining physical functioning	Contact with family and friends	Attending day centre – passive
	Obtain a sense of normality	Home care – a source of contact	Participating in activities

### The Need to Be Physically Active and Spend Time Outdoors

The participants described being physically active as important for maintaining their legs and being able to ‘stand on their feet’. Some walked several hours every day, which increased their well-being, while others were not able to keep up their walking speed anymore because they had been less active during the COVID-19 pandemic. Several stated that their memory loss interfered with their ability to be physically active. One of the biggest challenges of being able to be physically active was the fear of going out on their own because they had difficulties finding their way home again, as described in the following quotation:

*I like to be out in the nature – I like that but I’m afraid to go alone because I easily get lost. Don’t have any sense of direction anymore, and therefore it’s safer to be with somebody. (P3)*

While talking about how memory loss had impacted their ability to stay physically active and go out on their own, it seemed that it could be difficult to find somebody to accompany them on walks. Most of the participants had regular physiotherapy, and they would like the physiotherapist to go with them for a walk rather than doing exercises. However, when they asked the physiotherapist to go for a walk with them, they were told that this was not something that the physiotherapist could offer. They received the same answer when they asked if the home care staff could go for a walk since the home care service is restricted to offering specific physical help in the home, such as assisting with personal hygiene and preparing food. Since it was difficult to find someone to accompany them on walks, some participants had started to attend an exercise program organised by one city district in Oslo. However, even though they could explain the rationale for signing up for an exercise programme, some were not sure how often they were going, and one had forgotten to go, as expressed in the following quotation:

*I should have been there but couldn’t – don’t know what happened – think I forgot it. (P2)*

While talking about the need to be physically active, the participants described different motivations. They wanted to maintain their independence and continue to live at home as long as possible. One participant emphasised that even though she wanted to stay at home as long as possible, the day might come when she would need to go to a nursing home. Another motivation to stay physically active was the positive impact they thought it could have on their cognitive functioning. One participant who had difficulties expressing herself during the conversation commented:

*I think fresh air helps the brain a bit too. I got to try a little bit, don’t want everything to disappear. (P2)*

Several other barriers made it difficult to stay physically active outside their home. The fear of falling and having experienced falling outside their apartments made some afraid of going out on their own. One said that she struggled with dizziness, while others had experienced a general physical decline that made it difficult to move around, as in the following quotation:

*I can hardly move so I sit in a chair a lot, and that’s stupid, because I’m supposed to move. But I get so dizzy, and then I must sit down. (P1)*

Despite having some insight into their situation and the reasons that they had difficulties being physically active, some spoke about their plans, hopes and dreams for the future, while others were more occupied with how they could be more physically active. One participant said that she was considering buying a bicycle since the one she had was very old, while another participant stated that she was considering moving to another place because of the hills around her current home. Nevertheless, at the same moment, she expressed that moving to another place would be traumatic for her and that she would lose the beautiful view over the fjord.

### The Need to Staying Socially Connected

All the participants described their families as kind, helpful and important in giving meaning to their daily life. Receiving visits from their children and grandchildren was important for most participants, especially when their visitors took the time to have a meal with them. One participant commented:

*They stop by once in a while and have dinner with me, and I sometimes invite them out for dinner as well. (P6)*

Even though they appreciated these visits from family members, they also valued the practical help they received from family and friends, such as help with shopping or bringing food. Nevertheless, there was some variation in how much contact they had with family members. Some were conscious that their children had their own lives and that they could not expect them to come too often, while others were concerned about being a burden to their children. One participant said:

*He [her son] lives not too far away, and I see him, but eventually it gets a little bad because he has to take responsibility for me. I don’t want to do that because I think it eats away in a way. (P6)*

One was very grateful that she had a boyfriend, a person to be close to with whom she could spend time, instead of being alone all the time. However, this participant expressed that the surroundings were unfamiliar. She said she did not know the roads or the people who lived in the other apartments. Despite the contact with family, friends and neighbours, the participants seemed to spend a lot of time on their own. Consequently, the days could be long and boring since they did not have anything to do, as in the following statement:

* I’m just sitting her, doing little…alone. (P2)*

While all the participants appreciated visits from family and friends, they stressed that daily visits from the home care service were very important. Some expressed that the visits from the home care service prevented them from feeling lonely and were essential in their daily life to keep them safe and give them confidence that somebody would find them if they got ill or fell:

*I fell and almost killed myself. If they hadn’t found me then, I would have been done. For there were many hours I was just lying there. I was sick, too. I was going to the toilet, then everything became black afterwards. By then, I had fallen off the toilet. I had a home care nurse, and if they hadn’t found me, I would have been done. (P6)*

In addition to getting practical help from the home care service, several stated that they would like to have more time to talk to the home care staff. However, these participants were conscious that the home care staff had much to do and were often in a rush to get to the next client. When probed about how they experienced this ‘rush, they defended the home care staff and were concerned about bothering them, as in the following comment:

*The only thing I miss is that suddenly they’re going to be somewhere else, so they don't come. Because you love them so much, you know. (P1)*

Participants felt sorry for the home care staff and emphasised that they were all ‘nice and caring ladies’ and excused them for sometimes being late or not showing up.

### The Need for Meaningful Activities

Most of the participants spent the day at home, passing the time by watching TV or reading newspapers or books. They often felt bored and lacked the initiative to find something to do. When asked about what TV programme they preferred to watch, some were not able to mention a specific programme. However, for other participants, watching TV programmes was not important but rather something that filled gaps in their day, as follows:

*Either it has to be a programme that interests me to make the time pass, or it has to be something that, I keep saying, I can learn from, but it doesn’t help much because I forget it the next moment. (P2)*

When talking about what gave their daily lives meaning, several shared how they had been active earlier in their lives, working and travelling to different countries. Their present situation with little to do contrasted greatly with their earlier life; however, talking about this seemed to bring positive memories, as in the following comment:

*Yes, I have travelled a lot. I have a bunch of them [friends], but that’s because I’ve had friends to travel with… I don’t travel that much now. (P4)*

With regard to searching for new meaningful activities, one participant said that she used to go for long walks to pass the time. For some, seeing an art exhibition or going to the theatre, organised by the home care service, family or friends, gave meaning to daily life. In contrast, others were happy with whatever was offered, and it could be enough to just get out of the house for a while.

*I have shown up when there have been opportunities. And I go out alone and just look at people. So, I’m not dependent on talking to anyone. (P8)*

Although they spent most of their time at home, participants also attended a day care centre. While some shared that they attended once or twice a week, others were not sure how often they attended. However, several said that the day care centre was boring at times since the activities offered, such as throwing balloons at each other, were experienced as meaningless. Such activities did not require much physical movement, and in general, they would have liked to have more physical activities. They referred to participants at the day care centre who often slept in a chair after having breakfast and commented that most of the day consisted of eating and sleeping, as in the following statement:

*There’s not much to do. There you sit and sleep a lot. We also eat, maybe sleep a little, too. Then we probably were awake again…It’s the same again and again. (P7)*

Overall, the participants felt that the activities organised by the home care service offered positive experiences that gave them a pleasant break from what they described as long days at home. These activities with friends and other people were stimulating and helped them pass the time. Having the opportunity to go out occasionally was mentioned as positive, and some said they had not been out for years. They emphasised that being able to see other people, talk ‘properly with somebody’ and get to see new places that they would not have been able to go to on their own was valuable:

*Anything, because I’m just sitting here [at home] like a dead person. That’s why I go for a walk so I can use my legs, at least. (P2)*

When asked what kinds of activities they would like to attend in the future, only a few were able to express their wishes. Activities such as art and theatre were mentioned, but overall, participants stressed the importance of having something to do instead of spending the whole day at home.

## Discussion

The aim of this study was to describe what women with dementia who receive homecare services consider important in their daily lives. The data analysis identified three categories: The need to be physically active and spend time outdoors, The need to staying socially connected and The need for meaningful activities.

The participants emphasised the importance of being physically active in their daily life, and some reported that they walked hours every day, which increased their well-being. Being physically active and going for walks is part of Norwegian culture, and enjoying physical activity may make PwD feel better and promote emotional well-being (Cedervall et al., 2015; Farina et al., 2021). Identity is closely tied to what we do (Cedervall & Aberg, 2010), and being physically active may be a way to confirm one’s identity (Telenius et al., 2022). Being physically active could also contribute to a sense of self and personal feelings of wholeness (Goins et al., 2015). Physical activity is something that PwD can do as part of their daily routine that could allow them to be engaged with the world (McDuff & Phinney, 2015). Daily walks could make PwD feel healthy and fit (McDuff & Phinney, 2015), give meaning to their daily life (Telenius et al., 2022) and be a means of passing the time (McDuff & Phinney, 2015). Being physically active may also have been a coping strategy for the participants because the days were long, and they had little to do. However, PwD may struggle with organising their time and may need structure in their daily life (McDuff & Phinney, 2015). Being physical active seemed to be important for the participants to maintain their cognitive function. Similar findings were reported in a study that found that engaging in physical activities was meaningful to community-dwelling PwD since they believed that these activities would improve memory and restore their cognitive abilities (Han et al., 2016). Identifying the underlying motivation for an individual with dementia to engage in different activities is in line with person-centred care approach and important to tailor actives that are that are meaningful for the individual (Han et al., 2016; Marulappa et al., 2022). However, in line with previous research (Chong et al., 2014; Moe et al., 2021; Telenius et al., 2022), participants in this study experienced several barriers to being physically active, including being dependent on others to be physically active, fear of falling, difficulties with directions and concerns about getting lost. Other barriers to physical activity for PwD include having reduced eyesight, pain, lack of energy, fatigue, incontinence, and memory problems (McDuff & Phinney, 2015). Older women living alone appear to have poorer health outcomes such as reduced independence and higher falls risk, unmet care needs and lower mood, self-esteem, and life satisfaction compared to those living with family (Forward et al., 2022). This may also influence the ability of being physically active.

Participants described family members as kind, helpful and an important source of meaning in their daily lives. Visits from family members, especially having a meal together, was appreciated. For older people living alone, support and relationship with their children may provide a “network of belonging” and a sense that “they matter to someone” (Soulières & Charpentier, 2022). People who experience positive support from family and friends may experience their lives as more meaningful and valuable than those who lack such support (Eriksen et al., 2016; Krause, 2007). Family members may be motivated to keep a relative with dementia at home because, through knowledge of the person, they are attuned to their needs and preferences (Marulappa et al., 2022). Therefore, family members should be included in care planning to optimize care and support. In this study, the frequency of contact that the women had with their family members varied. However, *how* a person experiences the quality of a relationship may be more important than the number of social connections they have (Shim et al., 2012, 2013). In line with a previous study (Eriksen et al., 2016), participants in this study seemed to be protective and conscious that their children had their own lives, and they were concerned about being a burden. Furthermore, participants described the daily help that they received from the home care service as important. These visits prevented them from feeling lonely, and the home care staff were described as kind, helpful and essential in keeping them safe. In contrast, previous research described a more nuanced experience with home care services. One study found that some older people experienced uncertainty because they dealt with many different home care staff who were not always able to help, while others were pleased with the home care staff and the way they interacted with older people (Moe et al., 2021). However, participants were aware that home care staff had heavy workloads and often rushed to reach the next client. A perceived lack of time and control among health care professionals is an important barrier to person-centred care (Marulappa et al., 2022). Although home care visits are often short and task-oriented, they may brightened up the day and provide PwD a sense of safety (Fæø et al., 2020).

Most of the participants attended day-care centres, but they reported few meaningful activities and feeling bored since they were just sleeping and eating. These experiences are inconsistent with a person-centred care approach which emphasize empowering individuals with dementia to make independent choices and participate in decisions about their care and activities (Edvardsson et al., 2008; Kitwood, 1997; McCormack & McCance, 2010). In line with a person-centred care approach (Marulappa et al., 2022), day-care centre staff should understand each woman with dementia’s needs and promote autonomy by engaging her in meaningful activities. Day-care programs must be tailored to individuals’ needs, interests (Chong et al., 2014; Lindelöf et al., 2017; Malthouse & Fox, 2014) and functional level (McCormack & McCance, 2010; Norwegian Directorate of Health, 2017). However, limited knowledge of person-centred care and low perceived status within organizations and resource constraints are important barriers to implementation of a person-centred care approach (Marulappa et al., 2022). Furthermore, participants’ experiences of attending a day-care centre may reflect a desire to remain as physically active as they were before diagnosis. Predictable, familiar activities help PwD to retain a sense of purpose and control (Nygård, 2004; Roach & Drummond, 2014). The participants had mild to moderate cognitive impairment, which may have influenced their responses; people with lower awareness can report higher quality of life scores than with greater awareness (Strandenaes et al., 2022). Contrary to the findings of this study, previous studies have reported that PwD enjoyed the day care centre because they provided physical activities that gave meaning, structure and rhythm to their day (Telenius et al., 2022), and benefits for physical functioning, cognition and social stimulation (Strandenæs et al., 2018).

For some participants, activities organised by home care services and contact with family members or friends gave meaning to daily life, while for others, simply leaving the house, taking a break from routines and passing the time was important. The social activities offered by home care services appeared to be tailored to participants’ needs and to create a stimulating social environment consistent with a person-centred care approach (Brooker & Lathan, 2016). These activities may have been meaningful by helping participants avoid being or feeling alone, fostering belonging through participation itself rather than through the shared experience of dementia (Han et al., 2016). Previous research indicates that, for older people living alone, activity participation serves an important social function beyond entertainment or identified needs (Soulières & Charpentier, 2022). Caregivers of PwD have similarly highlighted stimulation as a reason why activities are meaningful, noting their potential to support learning, cognitive and emotional health, and to alleviate boredom (Roland & Chappell, 2015). The emerging focus on dementia-friendly societies should encourage new ways of including women with dementia in everyday life. Nurses may promote autonomy and independence by engaging PwD in meaningful activities (Marulappa et al., 2022). Within a person-centred care approach, nurses should acknowledge that individuals can experience life and relationships despite dementia and, consequently, should offer and respect choices (Edvardsson et al., 2008). Participants who were able to express their future wishes emphasised art, theatre, and opportunities to do more than simply share a meal. Consequently, nurses need to recognise that experiences of meaning vary between PwD (Holst & Hallberg, 2003). This aligns with the principles of person-centred care described by Kitwood and the VIPS framework, which emphasises valuing people (V), an individualised approach (I), the perspective of persons living with dementia (P) and social engagement (S) (Brooker & Latham, 2015; Kitwood & Bredin, 1992).

### Strengths and Limitations

The second author (IH) holds a degree in psychology and a PhD in dementia care and the last author (BSS) is a registered nurse with a PhD in dementia care and extensive clinical experience caring for PwD. Both authors have experience in communicating and interviewing PwD. The first author is a professor and registered nurse with expertise in qualitative research; the third author is an occupational therapist: and the fourth author is medical doctor and professor with extensive expertise in dementia care and geriatric psychiatry.

The interviewers’ expertise in, and experience of, communicating with PwD were important for facilitating conversation during the interviews, thereby enhancing the credibility of the findings. Furthermore, the research team’s diverse clinical and research experience contributed valuable perspectives to the data analysis. Dependability was enhanced by the fact that the same two authors conduct all the interviews. Confirmability was enhanced by including quotations that brought forth the participants voices and illustrated that the interpretations were grounded in the data. Transferability was enhanced by providing rich descriptions of the method, the participant characteristics and the setting, and the quotations that support the findings. A strength of the study is that the participants were recruited from two different city districts, which contributed to diverse experiences.

A limitation may be that data on participants’ functional status and comorbidities were not collected, which would have helped readers contextualize the findings. Ultimately, researchers, nurses and other professionals must assess whether the findings are relevant to their context. Another limitation is the small sample size. However, participants spoke openly during the interviews and shared varied accounts about daily life, providing sufficient information power (Malterud et al., 2016). Moreover, data richness and transferability do not necessarily increase by include a larger number of participants (Graneheim et al., 2017).

Although the activities organised by the home care service target both men and women, only women participated in this study; PwD living in rural areas and men may have experiences that differ from those this study identified. Furthermore, as participants were recruited through a gatekeeper and were already engaged in organised activities, there is a potential selection bias. Interviewing PwD is challenging: participants sometimes had difficulty following questions and probes and changed topics, which may have limited the data in terms of the experiences and nuances that this study was able to identify. Despite these limitations, this study provides an understanding of the world from the perspective of women with dementia who receive home care services.

### Implications for Practice and Research

Home care nurses, managers and nurse educators should recognise the importance of enabling women with dementia who receive home care services to be physically active and to spend time outdoors by supporting outdoor activity or organise group exercise programmes tailored to their needs. Regular visits from home care staff may be important for preventing loneliness and promoting a sense of safety and security at home. Increasing relational time during these visits may help these women continue living at home. The findings also underscore the need for a person-centred care approach in home care services and day-care centres, in which staff tailor activities so that women with dementia perceive them as meaningful.

This study contributes to nursing science by describing what women with dementia who receive home care services consider important in their daily lives, as this population remains understudied. Future studies should examine what additional help and support are needed to enable these women to continue living at home for as long as possible.

## Conclusion

This study provides insight into what women with dementia who receive home care services consider important in their daily lives. These women may value being physically active, for example by walking. Contact with family and friends, and visits and support from the home care service, may also be important to them. However, the activities offered by the day care centre did not meet their needs. The need for meaning in daily life may be met in different ways, and it is important to tailor activities for women with dementia.

## Supplemental Material

Supplemental Material - What Women with Dementia Who Receive Home Care Services Consider Important in Daily Life: A Qualitative Study Supplemental Material for What Women with Dementia Who Receive Home Care Services Consider Important in Daily Life: A Qualitative Study by Simen A. Steindal, Ingebjørg Haugen, Orla Brady, Knut Engedal and Benedicte Sørensen Strøm in Sage Open Nursing.

Supplemental Material - What Women with Dementia Who Receive Home Care Services Consider Important in Daily Life: A Qualitative Study Supplemental Material for What Women with Dementia Who Receive Home Care Services Consider Important in Daily Life: A Qualitative Study by Simen A. Steindal, Ingebjørg Haugen, Orla Brady, Knut Engedal and Benedicte Sørensen Strøm in Sage Open Nursing.

## Data Availability

The data is not available. Our research data includes sensitive information.*
