# Supplementary material for: What Women With Dementia Who Receive Home Care Services Consider Important in Daily Life: A Qualitative Study
Source: SAGE Open Nurs. 2026 Aug 17;12:23779608261479697. doi: 10.1177/23779608261479697 (PMC13487074; doi:10.1177/23779608261479697)
Supplement: Supplementary file 2 [file sj-pdf-2-son-10.1177_23779608261479697.pdf]

Supplementary File 2 Interview Guide

Theme 1: Interests and participation in different types of activities

How do you spend your day now?

What types of activities did you take part in before the pandemic?

To what extent have you been able to continue with these activities during the pandemic?

What types of activities are you participating in now?

Theme 2: Experiences with taking part in the activity

Which of the activities organised by the home care services have you taken part in?

What motivated you to participate in the activity?

Can you tell me a bit about how you experienced taking part?

- Inhibiting and facilitating factors

- What worked well? What was challenging?

- Is there anything that could have been done differently?

- Group size

- Time of day/duration

What did these activities mean to you?

Theme 3: Wishes after the end of the project period

Would you like to continue with any of the activities?
